# Calming The Storm By Sympatholysis

**Published:** 2011-03-25

**Authors:** Johnson Francis, Tara Bourke, Kalyanam Shivkumar

**Affiliations:** 1 KMCT Heart Insitute & Research Centre, KMCT Medical College, Calicut, Kerala, India; 2 UCLA Cardiac Arrhythmia Center, Ronald Reagan UCLA Medical Center, David Geffen School of Medicine at UCLA, Los Angeles, CA 90095, USA

**Keywords:** electrical storm, sympathetic chain blockade, thoracic epidrual anaesthesia, stellate ganglionectomy

An electrical storm is a fearful and life threatening experience for the patient while it is a therapeutic challenge of the extreme degree for the physician. In this issue of the Journal, Proietti and Sagone have presented an in depth discussion on the various aspects of the electrical storm including the current day management [1]. As rightly mentioned by them, the present definition of an electrical storm would be the occurrence of three or more episodes of ventricular tachycardia or ventricular fibrillation in 24 hours, requiring either anti-tachycardia pacing or a shock. This editorial note will focus on the management of a refractory electrical storm with sympatholytic measures, other than the conventional pharmacologic beta adrenergic blockade.

The concept of sympathetic blockade for the management of an electrical storm is not new as intravenous beta-blocker therapy is a well known therapeutic option in this setting [2]. Similarly left cardiac sympathetic denervation (LCSD) has been in use for prevention of the sympathetically driven life threatening arrhythmias in congenital long QT syndrome and catecholaminergic polymorphic ventricular tachycardia [3,4]. LCSD has also been shown to contribute to a reduction in the incidence of sudden cardiac death among subgroups of post myocardial infarction patients at high risk [5]. Mahajan  et al initially reported the use of thoracic epidural anaesthesia (TEA) for the control of an electrical storm [6]. Bourke et al from the same institution have further studied the efficacy of both LCSD and TEA in the management of electrical storms [7]. These measures were considered only when the storm or malignant ventricular arrhythmia were refractory to all conventional measures including intra venous beta-blocker therapy, antiarrhythmic drugs, device reprogramming to minimize shocks, treatment of reversible causes  such as myocardial ischemia and electrolyte disturbances, deep sedation and intubation with induction of general anaesthesia where necessary and catheter ablation when feasible. TEA was planned if the surgeon performing LCSD was not available or the patient was awaiting cardiac transplant or catheter ablation. Otherwise an LCSD was preformed, which is a more definitive therapeutic option [7].

Thoracic epidural anaesthesia involves application of local anaesthetic directly onto the sympathetic chain which results in almost immediate sympatholysis. TEA was given via an epidural catheter placed at the T1-2 or T2-3 interspace via a paramedian approach. Intrathecal or intravascular placement was excluded by the lack of aspiration of cerebrospinal fluid or blood. Bupivacaine was the agent used to obtain the anaesthesia and subsequent antiarrhythmic response, a 1ml bolus of 0.25% Bupivicane followed by an infusion at a rate of 2ml/hour was administered [7]. The effects of TEA on hemodynamic parameters including heart rate, mean arterial pressure, cardiac index and central venous pressure are minimal [8]. TEA was well tolerated by all the patients in their series in which 6/8 patients had a greater than 80% reduction in arrhythmia burden following TEA. No adverse effects were reported  [7].

LCSD was done by a video-assisted thorascopic approach (VATS). The pleural cavity was entered through three small incisions in the left subaxillary area; a double lumen endotracheal tube allowed ventilation of the contralateral lung while the ipsilateral lung was deflated and non ventilated to facilitate access to the sympathetic chain. Resection of the lower half of the stellate ganglion, along with T2-4 ganglia of the sympathetic chain was performed [7]. Patients in this series had diverse aetiologies of cardiomyopathy including ischaemic and nonischaemic, sarcoid, ARVD and hypertrophic. Five out of nine patients with refractory ventricular arrhythmias had a complete or partial reduction in arrhythmia burden following LCSD with 7/9 patients surviving to hospital discharge. Complications in this series included one partial Horner's syndrome which resolved after 6 months, an apical pneumothorax (which was managed conservatively) and transient facial paresthesia occurred in one patient. No procedural deaths occurred. The safety and feasibility of VATS LCSD has been reported in several small series previously [9-12]. Atallah J et al [12]  used VATS left cardiac sympathetic denervation for treating children with intractable ventricular arrhythmias. Four each of their patients had long QT syndrome and catecholaminergic polymorphic ventricular tachycardia while one had idiopathic ventricular tachycardia.

Another older approach to the problem of electrical storm is a left stellate ganglionic blockade (LSGB). LSGB can be given percutaneously by an anterior approach between the trachea and the carotid artery, within several millimeters anterior to the lateral process of the spine. The blockade was done using 1% xylocaine which was injected until a Horner's syndrome or partial Horner's syndrome developed [2] however repeated dosing and inability to use concurrent anticoagulation are limitations in the clinical setting.

Sympathetic hyperactivity is an important modulator of ventricular arrhythmias, including electrical storm [13] therefore neuraxial modulation is an attractive option for arrhythmia management. These treatment modalities can be considered when standard treatments fail, they may be used as a bridge to cardiac surgery or catheter ablation procedures or may be used as definitive therapy when revascularization, transplantation or catheter ablation procedures are not feasible. Large prospective randomized studies are needed to further define the clinical role of these therapeutic strategies in the future.
